# Pericardial Effusion-Associated Hyponatremia

**DOI:** 10.1155/2020/8847666

**Published:** 2020-07-13

**Authors:** Ahmed A. Hanfy, Nicholas T. Manasewitsch, Bryce D. Beutler, Daniel Antwi-Amoabeng, Mohamed Elnaggar, Munadel Awad, Gurpreet S. Chahal

**Affiliations:** Department of Internal Medicine, University of Nevada, Reno School of Medicine, Nevada, USA

## Abstract

Pericardial effusion has been identified as a rare cause of hyponatremia. In most patients, pericardiocentesis results in rapid correction. We describe a 67-year-old male who presented with pericardial effusion-associated hyponatremia secondary to cardiac resynchronization therapy-D placement that resolved following evacuation. In addition, we review the literature on pericardial effusion-associated hyponatremia.

## 1. Introduction

Hyponatremia is defined as a serum sodium concentration below 135 mEq/L. It is most commonly asymptomatic but may manifest with headaches, nausea, seizures, and death due to cerebral edema [1].

Pericardial effusion has been identified as a rare cause of hyponatremia. Interestingly, pericardiocentesis often results in rapid and complete reversal of hyponatremia [2–9]. The mechanism underlying pericardial effusion-related hyponatremia remains to be established. However, it has been hypothesized to result from increased antidiuretic hormone release in the setting of decreased free water excretion [2, 5, 8].

We describe a 67-year-old male with pericardial effusion-associated hyponatremia who experienced rapid and complete normalization of serum sodium following pericardiocentesis.

## 2. Case Presentation

A 67-year-old male with a history of nonischemic cardiomyopathy, non-insulin–dependent diabetes mellitus, and hypertension presented with progressively worsening dyspnea and cough of three weeks duration. Two months prior to presentation, the patient had undergone cardiac resynchronization therapy-defibrillator (CRT-D) placement for nonischemic cardiomyopathy and left bundle branch block with a left ventricular ejection fraction of less than 35%. No operative complications were reported.

**Figure 1:**
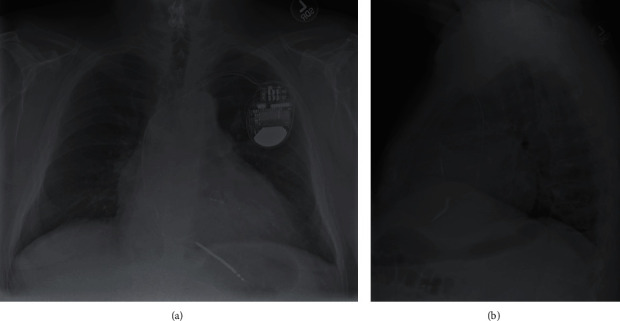
A two-view chest X-ray demonstrating enlarged cardiac silhouette, minimal bibasilar atelectasis, and appropriate placement of pacemaker leads (a, b).

Vital signs were within normal limits (pulse: 84 beats per minute; blood pressure: 132/68 mmHg; temperature: 36.1°C; respiratory rate: 18; oxygen saturation: 94% on room air). Physical examination was significant only for bibasilar rales; no pericardial friction rub or elevated jugular venous pressure were appreciated, and there were no clinical signs or symptoms concerning for tamponade. Laboratory studies demonstrated hyponatremia (sodium: 123 mEq/L; corrected for hyperglycemia: 127 mEq/L), hyperglycemia (glucose: 260 mg/dL), and normocytic anemia (hemoglobin: 11.0 g/dL, mean corpuscular volume: 86.7 fL). N-terminal pro b-type natriuretic peptide (NT-proBNP) was elevated to 239 pg/mL, which was near the patient's baseline. Serum potassium, bicarbonate, blood urea nitrogen, and creatinine were within normal limits. Electrocardiogram (ECG) showed an atrial-paced ventricular rhythm consistent with prior pacemaker placement. Two-view chest radiography revealed an enlarged cardiac silhouette, minimal bibasilar atelectasis, and appropriate placement of the pacemaker leads (Figure 1).

The patient was started on a fluid restriction of 1500 milliliters per day for hyponatremia and suspected heart failure exacerbation. However, subsequent laboratory studies showed significant worsening of the hyponatremia; by the second hospital day, the patient's serum sodium had decreased from 123 to 119 mEq/L (corrected for hyperglycemia: 123 mEq/L). Further investigation revealed serum blood urea nitrogen of 12 mg/dl, serum glucose of 264 mg/dl, calculated serum osmolality of 256 mOsm/kg, urine osmolality of 310 mOsm/kg H2O, and urine sodium of 42 mEq/L. Hypothyroidism was ruled out with a thyroid-stimulating hormone of 2.340 *μ*IU/mL.

**Figure 2:**
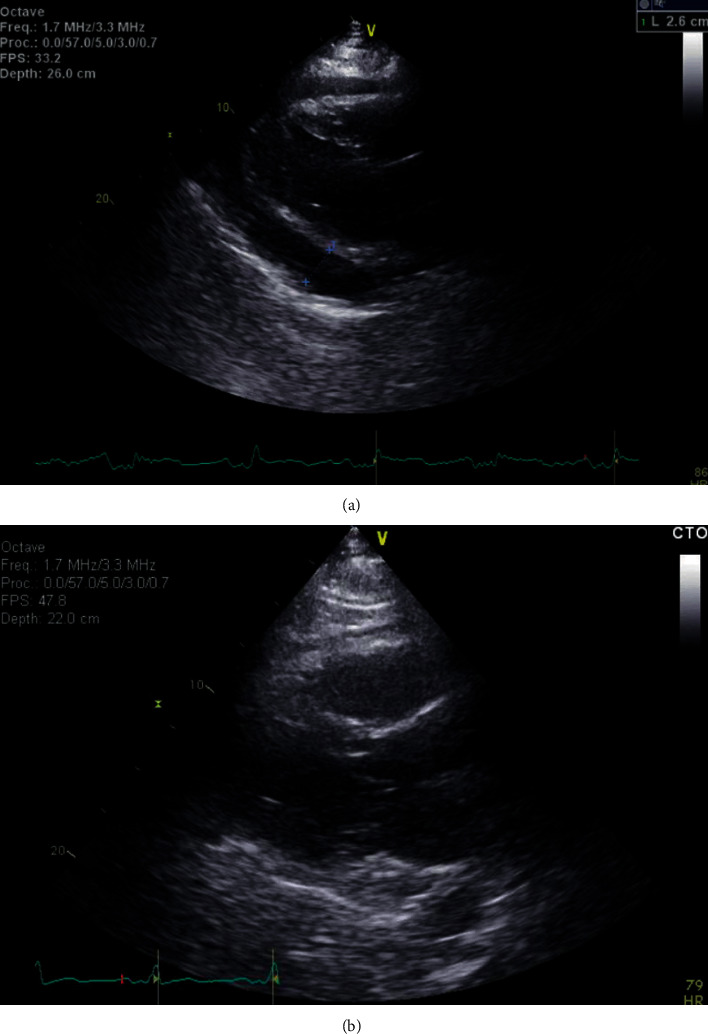
(a) Large circumferential pericardial effusion measuring 2.6 centimeters posteriorly, 1.5 centimeters anteriorly, and 1.8 centimeters at the apex with right atrial collapse and without right ventricular collapse. Significant inflow variation was noted across the mitral and tricuspid valves. Left ventricular ejection fraction was estimated at 55%. Mild concentric left ventricular hypertrophy was present. (b) Echocardiogram obtained the day after pericardial drainage demonstrates resolution of the pericardial effusion.

An echocardiogram demonstrated a large circumferential pericardial effusion with right atrial collapse (Figure 2). Left ventricular ejection fraction was estimated at 55% with normal diastolic function. Diastolic right ventricular collapse was absent. Echocardiographic features were consistent with early signs of cardiac tamponade. However, there were no clinical signs of tamponade and vital signs remained within normal limits.

**Figure 3:**
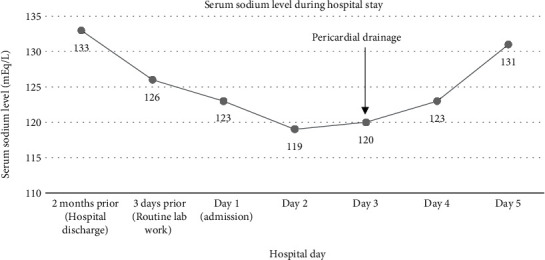
Serum sodium level during the hospital stay. Rapid correction of hyponatremia was observed within 36 hours of pericardiocentesis.

An urgent pericardiocentesis was performed with removal of approximately 1200 milliliters of sanguineous fluid. A follow-up echocardiogram was obtained one day after the procedure and demonstrated complete resolution of the effusion (Figure 2(b)). Serum electrolytes were monitored and showed a gradual improvement of the hyponatremia; serum sodium had increased from 120 to 123 mEq/L on the first postoperative day and to 131 mEq within 36 hours of the procedure (Figure 3). Calculated serum osmolality also increased to 278 mOsm/kg. Analysis of the pericardial fluid was negative for malignant cells and pathogens. Our patient's pericardial effusion was presumed to be secondary to his previous CRT-D placement. The patient was discharged home in stable condition on the third postoperative day. Outpatient follow-up four weeks later showed no recurrence of the pericardial effusion.

## 3. Discussion

Pericardial effusion represents a rare cause of reversible hyponatremia; this has been described in several case reports [2–6, 9] and two retrospective reviews [7, 8]. Our patient presented with progressively worsening hyponatremia in the setting of pericardial effusion with early tamponade and experienced rapid and complete normalization of serum sodium concentration following pericardiocentesis.

Resolution of hyponatremia following pericardiocentesis has been described in case reports by other authors [2–6]. In addition, in a recent review of 31 patients, Jong et al. found that individuals with cardiac tamponade-related hyponatremia demonstrated a statistically significant increase in serum sodium levels within 48 hours of pericardiocentesis [7]. Similar findings were reported in an article by Chang et al. [8]. A review of case reports that demonstrate improvement of hyponatremia following pericardiocentesis is summarized in Table 1.

The underlying cause of cardiac tamponade-related hyponatremia remains to be definitively established. However, a dual mechanism has been proposed: (1) increased cardiac pressure stimulates release of antidiuretic hormone with a consequent decrease in serum sodium from increased free water retention and (2) low cardiac output impairs renal free water excretion [2, 5, 8].

In our patient, malignant and infectious etiologies were ruled out based on pericardial fluid analysis. The pericardial effusion was presumed to be secondary to CRT-D placement two months prior. Postcardiac injury syndrome (PCIS), also called postperiocardiotomy syndrome (PPS), is well-documented following pacemaker placement and is a complication in 10-50% of patients [10, 11]. Pericardial effusion can develop between 5 and 56 days (mean: 21.5 days) after pacemaker placement [10]. Cardiac tamponade in PCIS is a rare phenomenon with an incidence of 0.1 to 0.6% [12]. Our patient was discovered to have a pericardial effusion 54 days following his pacemaker placement.

To our knowledge, there are only two reported cases of pericardial effusion-associated hyponatremia in the setting of pacemaker placement [9]. Rakhshan et al. described an 87-year-old woman and an 83-year-old woman in whom pericardial effusion-associated hyponatremia resolved following medical treatment with colchicine. Interestingly, in both of these cases, patients developed pleural effusion in addition to their pericardial effusion. Our case differs in that our patient had echocardiographic evidence of cardiac tamponade. Additionally, our patient was treated with pericardiocentesis rather than with colchicine, suggesting that medical and surgical management may both lead to resolution of hyponatremia.

The documented etiologies of pericardial effusion-associated hyponatremia include uremia [2], malignancy [5, 8], medication [6], and idiopathic [3, 4]. In each of these cases, patients had symptomatic cardiac tamponade with hemodynamic instability. Our case is unique in that our patient lacked clinical signs of tamponade; his only symptoms were cough and dyspnea. In addition, CRT-D placement represents a rare etiology of pericardial effusion.

## 4. Conclusion

Pericardial effusion-associated hyponatremia has been described in previous case reports [2–6] and retrospective studies [7, 8]. In most patients, hyponatremia resolves following medical management or pericardiocentesis. To the best of our knowledge, this is the first report of resolution of hyponatremia following evacuation of pericardial effusion secondary to CRT-D pacemaker placement. Our case report provides further evidence that treatment of a pericardial effusion rapidly corrects associated hyponatremia.

## Figures and Tables

**Table 1:** Summary of case reports illustrating resolution of hyponatremia following pericardiocentesis.

Author	Age/gender	Serum sodium level before treatment (mEq/L)	Serum sodium level after treatment (mEq/L)	Pericardiocentesis drainage	Time for resolution of hyponatremia
Present case (2020)	67/M	119	131	1500 mL	36 hours
Dalia et al. [2]	70/F	109	132	750 mL	48 hours
Weekes et al. [3]	49/M	95	134	2200 mL	14 days
Shafique et al. [4]	59/M	105	123	1700 mL	18 hours
Mouallem et al. [5]	58/F	126	139	1500 mL	1 day
Groves et al. [6]	29/M	124	131	1500 mL	36 hours

## Additional Points

*Take Away Lesson*. Hyponatremia is a rare manifestation of pericardial effusion. Serum sodium levels typically normalize following pericardiocentesis. Laboratory studies should be monitored closely in order to reduce the risk of hyponatremia-related complications in patients with an underlying pericardial effusion.

## References

[1] Lien Y. H. H., Shapiro J. I. (2007). Hyponatremia: clinical diagnosis and management. *The American Journal of Medicine*.

[2] Dalia T., Masoomi R., Sahu K. K., Gupta K. (2018). Cardiac tamponade causing severe reversible hyponatraemia. *BMJ Case Reports*.

[3] Weekes M. P., Reddi B. A. J., Wharton S., Gazis A. (2010). Hyponatraemia associated with cardiac tamponade and chronic fluid excess. *BMJ Case Reports*.

[4] Shafique R., Sarwar S., Wall B. M., Cooke C. R. (2007). Reversible hyponatremia related to pericardial tamponade. *American Journal of Kidney Diseases*.

[5] Mouallem M., Wolf I., Mindlin G., Farfel Z. (2003). Pericardial tamponade-associated hyponatremia. *The American Journal of the Medical Sciences*.

[6] Groves P. H., Shah A. M., Hutchison S. J. (1990). Hyponatraemia secondary to an inappropriately high release of antidiuretic hormone in cardiac tamponade. *British Heart Journal*.

[7] Jong B. H., Wei C. C., Shyu K. G. (2016). Improved hyponatremia after pericardial drainage in patients suffering from cardiac tamponade. *BMC Cardiovascular Disorders*.

[8] Chang F. K., Lee Y. C., Chiu C. H. (2012). Hyponatremia in patients with symptomatic pericardial effusion. *Journal of the Chinese Medical Association*.

[9] Rakhshan E., Mirabbasi S. A., Khalighi B., Khalighi K. (2015). Pericarditis-induced hyponatremia after cardiac electronic implantable device (CEID) procedures. *Am J Case Rep.*.

[10] Tsai W. C., Liou C. T., Cheng C. C., Tsai K. S., Cheng S. M., Lin W. S. (2012). Post-cardiac injury syndrome after permanent pacemaker implantation. *Acta Cardiologica Sinica*.

[11] Zeltser I., Rhodes L. A., Tanel R. E. (2004). Postpericardiotomy syndrome after permanent pacemaker implantation in children and young adults. *The Annals of Thoracic Surgery*.

[12] Miller G. L., Coccio E. B., Sharma S. C. (1996). Postpericardiotomy syndrome and cardiac tamponade following transvenous pacemaker placement. *Clinical Cardiology*.

